# Methods for engaging vulnerable and marginalized children through community based participatory research: a scoping review

**DOI:** 10.1186/s40900-025-00783-3

**Published:** 2025-08-27

**Authors:** Hayley Wagman, Nahid Iseyas, Zuhal Mohmand, Megan Watson, Sanjay Mahant, Victor Do

**Affiliations:** 1 Department of Pediatrics, University of Toronto, Hospital for Sick Children, Toronto, Canada; 2 Department of Pediatrics, University of Alberta, Edmonton, Canada; 3 3-557 Edmonton Clinic Health Academy, 11405 87 Ave NW, Edmonton, AB T6G 1Z1 Canada

## Abstract

**Background:**

Community-based participatory research (CBPR) is an evolving approach that fosters equitable partnerships between researchers and community members. However, significant knowledge gaps and a lack of consensus remain regarding effective methodologies and frameworks for engaging marginalized pediatric populations in CBPR. This scoping review aims to examine studies that explicitly state the use of CBPR to engage marginalized pediatric populations, with a focus on understanding the methodologies employed, effectiveness and identifying key lessons learned.

**Methods:**

A comprehensive literature search was conducted across four databases: Ovid MEDLINE, EBM Reviews, APA PsycINFO, and Web of Science. The review followed the Arksey and O’Malley framework and adhered to a protocol developed using the Preferred Reporting Items for Systematic reviews and Meta-Analyses (PRISMA) extension for scoping reviews. Studies were included if they focused on engaging vulnerable and marginalized pediatric populations through CBPR and provided detailed descriptions of their methodologies. Multiple reviewers participated in all stages of the review and data extraction, with descriptive analyses conducted to examine methodological approaches.

**Results:**

Of the 8671 unique studies identified, 46 met the inclusion criteria. Most studies targeted engagement with racial and ethnic minority populations, with few adopting an intersectional approach to define populations of interest or tailor engagement strategies. The majority of studies were conducted in the United States and published within the last 20 years. Common engagement methods included community partnership development, employing focus groups and interviews, convening advisory boards, and electronic participatory techniques, but there was little consistency in how these methods were defined or implemented. Evaluation of the effectiveness of CBPR methods was notably lacking. Researchers also highlighted significant challenges, including historical mistrust, logistical barriers, and limited resources, in engaging marginalized populations.

**Conclusions:**

There is a critical need for more robust and standardized methodologies to engage marginalized pediatric populations in participatory research. We also need ongoing efforts centered on evaluating effectiveness. This review highlights existing gaps and barriers, providing insights to inform future research and the development of evidence-based frameworks for empowering and meaningfully involving marginalized communities in CBPR.

**Supplementary Information:**

The online version contains supplementary material available at 10.1186/s40900-025-00783-3.

## Abstract (plain language summary)

Community-based participatory research (CBPR) is an approach where researchers and community members work together equally. This approach makes sure that research is meaningful and useful to the community it aims to help. This is especially important for children and youth from marginalized backgrounds who are often left out of research. However, there is limited information on the best ways to involve children and youth from marginalized backgrounds in research so they can help find solutions to the problems that affect them. To learn more, we conducted a scoping review - a broad search of existing studies - to understand how this approach is being used with marginalized children. We paid special attention to how young people and their communities were included, what methods researchers used, and what challenges they faced. We found that most studies focused on engaging children and youth from racial and ethnic minority communities. Some of the common ways researchers worked with these groups included partnering with community organizations, holding focus groups or interviews, creating advisory boards, and using online tools. However, there was no clear or consistent application or use of these methods, and most studies did not evaluate how well they worked. Researchers also faced challenges like community mistrust, not having enough staff or funding, and dealing with complicated ethical and logistical issues. Overall, our review shows that methods where researchers work together with communities could benefit from better guidance on their application and use. This will help make sure that children and youth from marginalized backgrounds, who are often overlooked, can have a say in research that will impact their health and lives.

**Supplementary Information:**

The online version contains supplementary material available at 10.1186/s40900-025-00783-3.

## Background

Community-based participatory research (CBPR) is an evolving research approach that emphasizes developing meaningful and equitable partnerships between researchers and community partners [1, 2]. In health research, CBPR represents a shift away from traditional researcher-subject dynamics toward collaborative partnerships where communities are valued as equal partners in the research process [3]. By prioritizing long-term commitments, knowledge-sharing, and community empowerment, CBPR has the potential to bridge gaps between scientific and local communities, enhancing health outcomes. Effective CBPR practices can foster trust with communities, ensure the cultural relevance of research methods and interventions, and build community capacity to address health challenges [4].

Despite its growing prominence, CBPR’s application to more vulnerable and marginalized children remains underexplored [5]. These populations—which can include racial and ethnic minorities, low-income families, LGBTQ + youth, and children with disabilities amongst others—face unique health challenges rooted in systemic inequities. Historically, they have borne a disproportionate burden of health disparities [6, 7]. Factors such as historical mistrust of research institutions, limited access to resources, communication barriers, and cultural differences can further hinder research engagement with these groups [6, 7]. This highlights the need for research methodologies that prioritize genuine community engagement, emphasize community-driven solutions, and implement culturally sensitive, contextually relevant interventions [6, 7]. While there are many definitions of vulnerable and marginalized pediatric populations, for the purposes of this review, we will define these communities as “Children and adolescents who experience heightened susceptibility to adverse health outcomes due to systemic, social, economic, or environmental inequities. These populations often face barriers to accessing quality healthcare, education, and essential resources, stemming from factors such as socioeconomic disadvantage, discrimination, lack of legal protections, or geographic isolation.” [8–10].

Although CBPR has been shown to be broadly effective [11], there is less clear evidence about how it is most effectively applied with children, particularly those from vulnerable and marginalized groups. Furthermore, wide variations exist in how researchers characterize and implement CBPR [12], and there is a notable gap in the literature regarding best practices for engaging vulnerable and marginalized children in participatory health research [13, 14]. For example, while some studies claim to adopt CBPR principles, it is unclear to what extent these principles are fully realized, especially in studies involving children and their families. Further, from an implementation science perspective, we need to consider how to support adoption and integration of these methodologies more broadly amongst the pediatric research community.

As CBPR continues to gain traction in pediatric research, it is crucial to understand how it is applied to vulnerable and marginalized children. Key questions include: What are the specific CBPR methodologies being utilized with marginalized pediatric populations? How have researchers been assessing the effectiveness of these methodologies? What can we learn to refine and establish best practices for engaging these populations?

This scoping review seeks to address these questions by examining how CBPR methodologies are being used to engage vulnerable and marginalized children and their families in health research.

## Objectives

The primary objective of this review is to understand the scope and nature of CBPR to engage marginalized children and their families in health research. Secondary objectives include understanding how these methodologies are assessed and identifying best practices as perceived by both researchers and community partners. By filling this critical gap, we aim to advance efforts to meaningfully involve marginalized pediatric populations in research, thereby contributing to the development of more equitable and effective health interventions.

## Methodology

We conducted a scoping review following the methods described by Arksey and O’Malley and advanced by Levac et al. [15], We used the Preferred Reporting Items for Systematic reviews and Meta-Analyses extension guidelines for scoping reviews (PRISMA-ScR) in our process and our reporting conventions.

### Identifying relevant studies

A literature search was conducted from inception until the date of searches (initially done August 2022, updated November 2024) with the assistance of a librarian from the Hospital for Sick Children Library Institute. Databases searched included Ovid MEDLINE (including Epub ahead of print, In-Process, In-Data-Review, and Other Non-Indexed Citations), EBM Reviews - Cochrane Central Register of Controlled Trials, APA PsychINFO, and Web of Science Core collection. We developed the search strategy iteratively as a study team. Some of the key terms used included: child, pediatrics, participatory, community based, marginalized, vulnerable, and health research. See Table 1 for Database search results and see Appendix A for the detailed search strategy.

**Table 1:** Database search

Database [Platform] Search last updated on	Results
Ovid MEDLINE(R) and Epub Ahead of Print, In-Process, In-Data-Review & Other Non-Indexed Citations and Daily	3270
EBM Reviews - Cochrane Central Register of Controlled Trials	775
APA PsycINFO	2124
Web of Science Core collection –	3426
TOTAL*not deduplicated	**9597**

*926 duplicates removed for a total included of 8671

### Review

Our review included three major components: title and abstract screening, full-text review, and data extraction. Covidence was used as a web-based review manager. All articles were reviewed in title and abstract screening and full-text phases by two team members, with conflicts resolved through consensus discussion at regular research team meetings. The research team consisted of five reviewers, with four reviewers paired into groups of two throughout the process. The fifth reviewer was present for all research meetings and led the group consensus discussions by reviewing the study in question as a group and ultimately deciding on a resolution if there was no group consensus. Please see Table 1 for the PRISMA protocol.

### Title and abstract screening

The selection criteria was developed and agreed upon by the study team. We regularly met as a team during the title and abstract screening to consider whether there needed to be revisions to our selection criteria. The following inclusion criteria was utilized for the review:

- Article must focus on marginalized paediatric populations.
  i. Marginalized populations include individuals and/or groups that experience discrimination and exclusion (e.g., social, political, or economic) due to relationships across economic, political, social, and cultural dimensions (This is to be defined by researchers, given that marginalization is context-dependent).
  ii. Paediatric populations (children and their caregivers) include ≤ 18 years of age (Articles can include individuals > 18 if the focus of the article remains on pediatric populations).
- Articles must describe their community-based participatory methodology in sufficient detail to allow for information extraction (e.g. would be excluded if they only state the use of community-based participatory research without describing its application and context).
- Articles must focus on health, medical and/or health systems.
- Only English-language articles were considered.
- All article types, including abstracts, conference proceedings, letters, editorials, and personal reflections were eligible, should they meet all other inclusion criteria.

Exclusion criteria:

- Article is not focused on marginalized pediatric populations (children or caregivers).
- Article does not self-identify as engaging in participatory research.
- Article is not focused on health, medical and/or health systems.
- Non-English articles, as we are not able to properly evaluate them.

We engaged in ongoing, robust discussions throughout the process to ensure the inclusion and exclusion criteria were both comprehensive and rigorous.

### Full text review

We held regular team meetings throughout the review process and ensured consensus before establishing the list of full-text review articles. We subsequently performed full-text review in duplicate to assess their eligibility for the data extraction phase of the review. We held regular consensus meetings to ensure consistency amongst the reviewers prior to finalizing the 46 articles that were included in the data extraction phase.

### Data extraction

Data extraction was performed in duplicate by two research team members. Disagreements were reviewed during research team meetings and discrepancies were resolved by consensus. Extraction was completed on a shared Excel document template, with categories for extraction developed and identified by the research team prior to initiating extraction. Broad categories included study demographics, participant demographics, comparators, study methodology, study outcomes, and details of engagement methods. Each large category had multiple subcategories.

### Data analysis and results synthesis

Descriptive analysis and statistics were employed to summarize the study findings. We utilized thematic synthesis to summarize the narrative findings from our data extraction process and we worked to present the results in summary tables and narrative summaries.

Of note, a compensated patient partner knowledge user who identifies with being from a structurally marginalized population was engaged during the review process for feedback on our process and findings. We held 3 separate meetings to discuss the methods, review collated results and for feedback on our analysis/discussion.

**Fig. 1:**
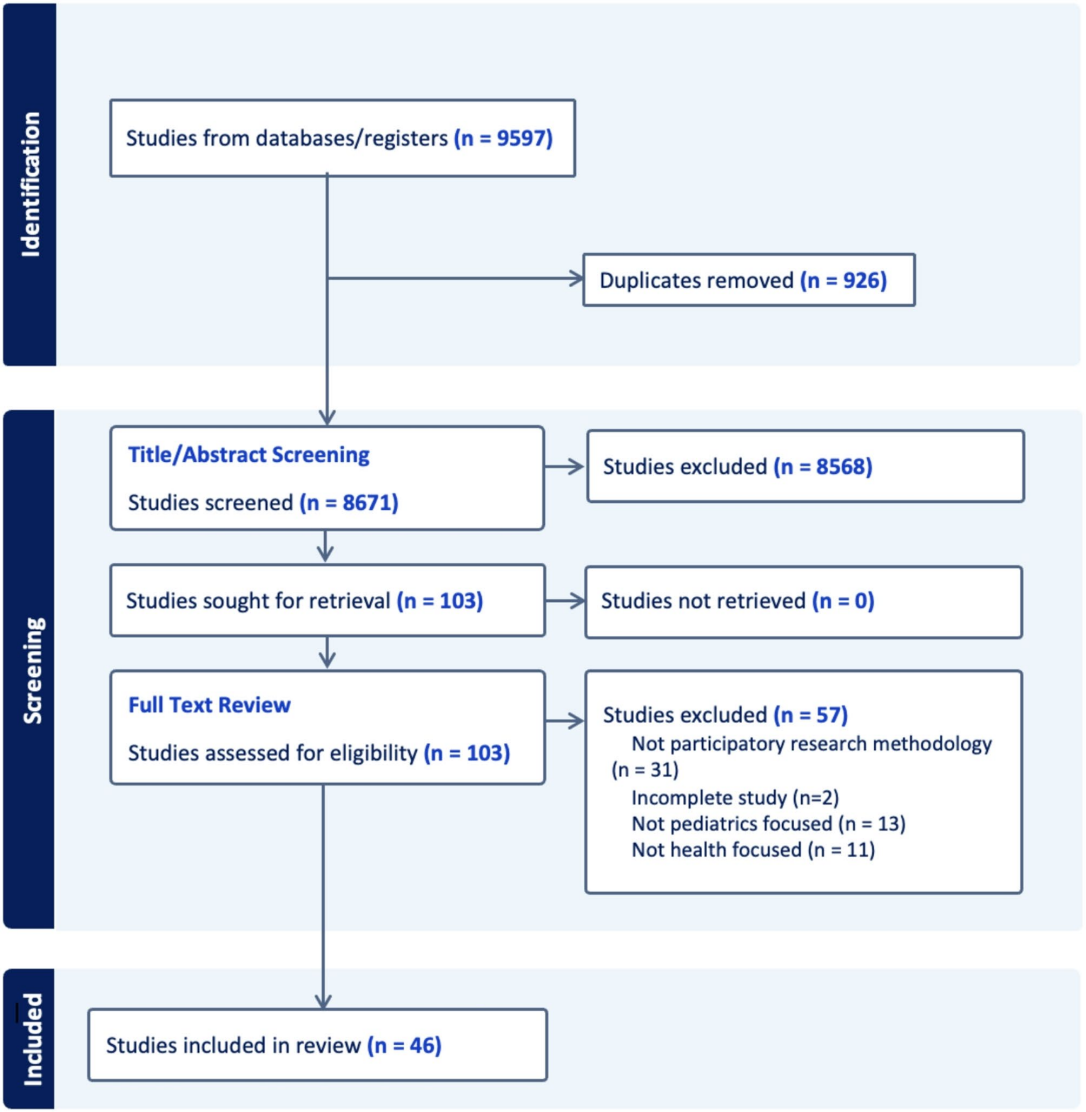
Protocol for scoping review

## Results

### Search yield

The search strategy yielded 9597 articles, of which 926 duplicates were removed, for a total of 8671 which underwent title and abstract screening. After title and abstract screening, 103 articles were selected for full-text evaluation. Following this review, 46 articles met the inclusion criteria for extraction. The remaining 57 articles were excluded for various reasons; 31 on further review either did not have enough details about methodology for extraction or did not actually note employing participatory research methodology, 13 were not pediatric focused, and 11 articles were not health-focused. 2 were incomplete studies with insufficient data for extraction.

### Article characteristics

The included studies were conducted in a number of different geographic regions, with the majority conducted in the United States (*n* = 27), followed by Canada (*n* = 6), Australia (*n* = 3), the United Kingdom (*n* = 2), Kenya (*n* = 1), Uganda (*n* = 1), China (*n* = 1), and four studies spanning multiple countries. Publications spanned three decades, with 4 studies published in the 2000s, 29 in the 2010s, and 13 in the 2020s. Study designs were predominantly mixed-methods [26], alongside a number of primarily descriptive studies [11]. Reviews that included articles and information around participatory research were included. Only two randomized controlled trials were identified. The studies addressed diverse health topics, including health promotion and engagement (*n* = 34), mental health (*n* = 8), chronic disease (*n* = 4), HIV prevention (*n* = 3), and sexual health (*n* = 2). Most studies considered racial and ethnic demographics as their defined vulnerable and marginalized population groups of focus (*n* = 31). See Table 2 for more details.

### Community engagement partners

Individuals/groups that were noted to be community partners formed the majority of those engaged in the studies included (*n* = 27). Community partners as a term was used by researchers to refer broadly to individuals, associations, and/or organizations who partnered with researchers in order to represent a broader group beyond themselves and who came from and/or worked with communities of interest. Other groups included healthcare professionals (*n* = 11), patients (marginalized pediatric patients) and/or caregivers (*n* = 10), non-patient youth (*n* = 10), governing organizations (*n* = 5), and school-based partners (*n* = 5). Nine studies involved other “community partners” that were not clearly defined or categorized.

### Pediatric populations engaged

The studies predominantly engaged marginalized youth pediatric populations aged 12 years and above. There were no studies identified that only sought to engage marginalized children and/or youth in the participatory design (e.g. all studies that involved youth did so alongside the engagement of families and caregivers). Direct engagement with younger children with marginalized backgrounds was limited in these studies (no study described in detail participatory engagement with children younger than 12, though 2 did describe methods to try to further include their perspectives), potentially reflecting the logistical and methodological challenges of incorporating younger age groups into participatory research. Table 3 includes more detailed information on populations engaged.

### Primary engagement methods

Studies employed a variety of engagement methods, often inconsistently defined. The most common approach described was the general use of “community partnership and relationship building” (*n* = 31), which focused on fostering collaborative relationships through various approaches including prioritizing shared decision-making, joint planning, and generally working to advance mutual ownership of research processes. The descriptions of these processes varied widely across the different studies. These partnerships involved diverse individuals, associations and/or organizations, including schools, faith-based groups, and community health clinics. 10 studies relied on focus groups, primarily for sharing information about research or gathering community perspectives to identify needs and priorities. Similarly, 9 studies used individual interviews, either informal or structured, to capture in-depth personal insights. Advisory boards, described in eleven studies, were more formalized partnerships designed to provide oversight and guidance for CBPR projects, with members including government representatives, community leaders, educators, and faith-based leaders. Photovoice, an innovative method empowering participants to document their lived experiences through photography, was used in seven studies to foster meaningful discussions and advocacy efforts. Table 4 provides further description of engagement methods.

### Engagement throughout the research process

Studies described partner participatory engagement most frequently during the study design and development phase, with 75% (*n* = 33) noting that this occurred. However, engagement declined in later study stages, with only 20% (*n* = 9) of studies involving partners in recruitment, 27% (*n* = 12) in data collection, and 38% (*n* = 17) in data interpretation and dissemination. Only 11% (*n* = 5) of studies reported involving partners throughout all stages of the research process, raising concerns about the depth and consistency of community engagement. This is illustrated further in Table 5.

### Core study team members

Limited information was available on the demographic and lived experience composition of the research study teams. Among the 23 studies that provided descriptive details of the study team, many did note including researchers who shared ethnic, linguistic, or cultural backgrounds with the target populations, which was thought to contribute to facilitating culturally relevant research. However, representation of other marginalized groups within research teams was sparse, highlighting an opportunity for more inclusive team composition.

### Theoretical frameworks

Theoretical frameworks guiding CBPR methodology in the research studies were inconsistently reported. Some studies 33% (15/46) reported that they had grounded their approaches in frameworks such as critical race theory, ecological systems theory, Indigenous paradigms, or decolonization methodologies. The majority of studies did not explicitly identify a guiding theoretical framework, potentially reflecting a gap in the methodological rigour and clarity of CBPR applications. For studies that did name certain frameworks, it was often not clear to what extent or how the work was grounded in the identified framework.

### Methodological evaluation

Nearly half of the studies (46%; *n* = 21) provided no formal assessment of their CBPR engagement strategies. Among the 25 studies that did describe some type of evaluation of their methodologies, a variety of techniques were described (some studies used multiple evaluation methods). Participant surveys (*n* = 5) were used to gather feedback on engagement experiences, while focus groups and interviews (*n* = 13) provided more in-depth insights into participants’ perspectives. Recruitment and retention rates (*n* = 7) were employed to assess the success of engagement strategies, and a few studies (*n* = 7) evaluated deliverables and outcomes to measure the impact of participatory methodologies on achieving research objectives. A notable limitation was the frequent conflation of engagement evaluation with assessments of intervention efficacy, which made it challenging to draw conclusions about the effectiveness of engagement methods. Table 6 details studies and their evaluation techniques with descriptions of the methodologies.

### Additional trends and challenges

Several challenges emerged across the studies. Historical mistrust of research institutions was a significant barrier frequently discussed, given that many of these populations of interest had experienced and continued to experience systemic inequities. Logistical and financial constraints further complicated the research process, with researchers citing insufficient resources and time affecting their engagement methodologies. Ethical and procedural challenges, such as navigating institutional ethics processes, were common, especially when employing novel participatory methods. Limited access to technology also posed barriers to recruitment and data collection. Despite these obstacles, studies highlighted the importance of sustained engagement, culturally sensitive methodologies, and flexibility in overcoming barriers to foster meaningful community partnerships.

**Table 2:** Article descriptive details

	# of studies	Studies
Year		
2005–2009	4	Flicker 2008, Savage 2006, Shalowitz 2009, Thomas 2009
2010–2014	13	Cruz 2014, Garcia 2012, Garwick 2010, Javier 2010, Lesser 2013, Liu 2011, Martin 2010, Shea 2013, Tanjasiri 2011, Tiwari 2014, Traub 2013, Vaughn 2013, Yonas 2013
2015–2019	16	Blanchet 2017, Green 2016, Jadwin-Cakmak 2019, Katz-Wise 2019, Langdon 2016, Livingood 2017, Merves 2015, Muhwezi 2019, Renzaho 2015, Shelef 2016, Shikako-Thomas 2016, Ungar 2015, Vindrola-Padros 2016, Willis 2016, Willows 2016
2020–2024	13	McCalman 2020, Raanaas 2020, Rocha 2022, Simpson 2022, Singer 2022, Teela 2022, Valdez 2020, Valdez 2021, Williamson 2022, Willis 2020, Yan 2021, Callejas 2022, Hamilton 2024
Country		
USA	27	Cruz 2014, Garcia 2012, Garwick 2010, Jadwin-Cakmak 2019, Javier 2010, Katz-Wise 2019, Langdon 2016, Lesser 2013, Livingood 2017, Martin 2010, Merves 2015, Rocha 2022, Savage 2006, Shalowitz 2009, Shelef 2016, Simpson 2022, Singer 2022, Callejas 2022, Tanjasiri 2011, Thomas 2009, Tiwari 2014, Traub 2013, Vaughn 2013, Willis 2020, Willis 2016, Yan 2021, Yonas 2013
Canada	6	Blanchet 2017, Flicker 2008, Shea 2013, Shikako-Thomas 2016, Ungar 2015, Willows 2016
Australia	3	McCalman 2020, Renzaho 2015, Hamilton 2024
United Kingdom	2	Vindrola-Padros 2016, Williamson 2022
Kenya	1	Green 2016
China	1	Liu 2011
Uganda	1	Muhwezi 2019
Multiple countries	4	Raanaas 2020 ^1,^ Teela 2022 ^2,^ Valdez 2020 ^3,^ Valdez 2021 ^4^
Study Design		
RCT	2	Cruz 2014, Tiwari 2014
Descriptive	11	Flicker 2008, Garcia 2012, Garwick 2010, Green 2016, Merves 2015, Shikako-Thomas 2016, Singer 2022, Thomas 2009, Willis 2020, Willis 2016, Callejas 2022
Mixed methods	26	Blanchet 2017, Traub 2013, Ungar 2015, Yan 202, Jadwin-Cakmak 2019, Javier 2010, Katz-Wise 2019, Langdon 2016, Lesser 2013, Liu 2011, Livingood 2017, Martin 2010, McCalman 2020, Muhwezi 2019, Renzaho 2015, Rocha 2022, Shea 2013, Shelef 2016, Hamilton 2024, Tanjasiri 2011, Teela 2022, Valdez 2021, Vindrola-Padros 2016, Williamson 2022, Willows 2016, Yonas 2013
Scoping Review	1	Raanaas 2020
Ethnography	1	Savage 2006
Literature Review	2	Shalowitz 2009, Vaughn 2013
Critical Review	1	Simpson 2022
Systematic Review	1	Valdez 2020
Health Topic ^		
Health Promotion and Engagement	34	Blanchet 2017, Cruz 2014, Flicker 2008, Katz-Wise 2019, Liu 2011, Livingood 2017, Muhwezi 2019, Renzaho 2015, Shalowitz 2009, Shikako-Thomas 2016, Simpson 2022, Singer 2022, Tanjasiri 2011, Thomas 2009, Tiwari 2014, Traub 2013, Hamilton 2024, Ungar 2015, Valdez 2020, Valdez 2021, Vaughn 2013, Vindrola-Padros 2016, Willis 2020, Willis 2016, Willows 2016, Yan 2021, Yonas 2013
Mental Health	8	Garcia 2012, Langdon 2016, McCalman 2020, Merves 2015, Raanaas 2020, Rocha 2022, Shea 2013, Williamson 2022, Callejas 2022
Chronic Disease	4	Garwick 2010, Martin 2010, Shelef 2016, Teela 2022
HIV Prevention	3	Green 2016, Jadwin-Cakmak 2019, Lesser 2013
Sexual Health	2	Javier 2010, Savage 2006
Marginalized Population ^+^		
Ethnicity/Race	Total *n* = 31 General minority − 5 Black − 4 Hispanic − 2 Latino − 6 Filipino-Americans − 1 Indigenous − 10 African-American − 8 Arab − 1 Pacific Islander − 1	Blanchet 2017, Cruz 2014, Garcia 2012, Garwick 2010, Javier 2010, Langdon 2016, Lesser 2013, Livingood 2017, McCalman 2020, Merves 2015, Raanaas 2020, Hamilton 2024, Renzaho 2015, Rocha 2022, Savage 2006, Shalowitz 2009, Shea 2013, Shelef 2016, Simpson 2022, Singer 2022, Tanjasiri 2011, Thomas 2009, Tiwari 2014, Traub 2013, Valdez 2020, Valdez 2021, Vaughn 2013, Willis 2020, Willis 2016, Willows 2016, Yan 2021, Yonas 2013
Location	Total *n* = 6 Rural − 4 Urban − 1 Industrial community − 1	Cruz 2014, Garwick 2010, Green 2016, Liu 2011, Muhwezi 2019, Rocha 2022
Sexuality	Total *n* = 4 LGBTQ + − 4	Flicker 2008, Jadwin-Cakmak 2019, Katz-Wise 2019, Valdez 2020
Low socioeconomic status (SES)	Total *n* = 11	Garwick 2010, Green 2016, Jadwin-Cakmak 2019, Martin 2010, Merves 2015, Muhwezi 2019, Shalowitz 2009, Shelef 2016, Tiwari 2014, Ungar 2015, Yonas 2013
Situational	Total *n* = 9 Immigrant − 3 Teen parents − 1 Refugees − 2 Homeless − 1 Street involved − 2 HIV high-risk − 2 Sex workers − 1	Blanchet 2017, Flicker 2008, Green 2016, Lesser 2013, Raanaas 2020, Renzaho 2015, Singer 2022, Traub 2013, Valdez 2020
Medical	Total *n* = 4 Cerebral palsy − 1 Chronic disease − 1 Disabilities − 1 Mental health − 2	Shikako-Thomas 2016, Teela 2022, Vindrola-Padros 2016, Williamson 2022, Callejas 2022

Details of studies included in full text review including year conducted, countries in which they were conducted, study type, and specific marginalized populations that were the focus

* Secondary design components include Qualitative, Longitudinal, Quasi-Experimental, RCT, Pragmatic Step-Wedge, Participatory Action, Cross-Sectional, Visual, ANGELO Framework

^ Total n is greater than 46 as some studied multiple health topics

^+^ Total n is greater than 46 as some involved multiple different vulnerable and marginalized population groups

^1^ USA, UK, Canada, Kenya, Uganda, Sierra Leone, Liberia, DRC

^2^ Netherlands, Canada

^3^ USA (*n* = 2), Canada (*n* = 1), Bosnia Herzegovina (*n* = 1)

^4^ USA, Mexico

**Table 3:** Specific ‘community partner’ groups engaged by researchers

Types of Partners	# of studies	Studies
Patients or caregivers	11	Garwick 2010, Green 2016, Liu 2011, Martin 2010, Muhwezi 2019, Shelef 2016, Singer 2022, Teela 2022, Ungar 2015, Williamson 2022, Hamilton 2024, Callejas 2022
Non-patient youth	10	Jadwin-Cakmak 2019, Javier 2010, Katz-Wise 2019, Langdon 2016, Livingood 2017, McCalman 2020, Merves 2015, Raanaas 2020, Singer 2022, Tanjasiri 2011
Community (individuals, associations, organizations)	28	Blanchet 2017, Cruz 2014, Cui 2019, Flicker 2008, Garcia 2012, Green 2016, Katz-Wise 2019, Langdon 2016, Lesser 2013, McCalman 2020, Merves 2015, Muhwezi 2019, Renzaho 2015, Rocha 2022, Savage 2006, Shalowitz 2009, Shea 2013, Singer 2022, Hamilton 2024, Tanjasiri 2011, Thomas 2009, Traube 2013, Ungar 2015, Valdez 2021, Willis 2020, Willis 2016, Yan 2021, Yonas 2013
Governing organizations (local, tribal, etc.)	5	Langdon 2016, Liu 2011, Martin 2010, Muhwezi 2019, Shalowitz 2009
Healthcare professionals	12	Green 2016, Katz-Wise 2019, Langdon 2016, Livingood 2017, Martin 2010, Merves 2015, Muhwezi 2019, Savage 2006, Shea 2013, Shelef 2016, Teela 2022, Hamilton 2024
Teachers/school-based	5	Garcia 2012, Green 2016, Langdon 2016, Liu 2011, Rocha
Other community partners	10	Blanchet 2017, Merves 2015, Muhwezi 2019, Raanaas 2020, Shalowitz 2009, Shelef 2016, Simpson 2022, Hamilton 2024,Teela 2022, Willows 2016

Descriptions of the types of groups of patients/community partners that researchers focused on engaging in each study.

**Table 4:** Primary engagement methods utilized

Methodologies	Description The articles described the methodologies as:	# of studies
Community Partnerships and relationship building	• Fostered collaborative relationships between research teams and community groups. • Emphasized joint planning, shared decision-making, and mutual ownership of research. • Included multiple different types of partnerships with local organizations such as immigrant settlement groups, faith-based organizations, cultural associations, schools, and community health clinics. • Regular meetings with community members to: ◦ Shape research goals and provide input. ◦ Assist with community outreach, especially during recruitment. ◦ Share personal stories, perspectives, and experiences. ◦ Co-develop solutions to health problems and build capacity. ◦ Involve community members as co-leads in research teams.	**33**
Focus Groups	• Often utilized with the stated objectives of gathering diverse community perspectives on specific issues relevant to community members. • Groups served to facilitate structured, yet open discussions among small groups. • Also utilized to foster collective understanding on research topics and help identify community needs and potential solutions to these needs based on lived experiences of participants.	**10**
Interviews	• Involved informal, semi-structured, and structured one-on-one conversations between community members and researchers. • Goal was often to create space for exploring detailed, personal insights and experiences regarding specific issues. Research teams aimed to capture authentic voices of the community which they hoped would enhance the relevance and impact of these studies. • Several studies had community leaders and healthcare workers help lead interviews in order to foster a sense of trust with community members.	**9**
Advisory Boards	• Often described as formalized academic-community partnerships that provide ongoing oversight and guidance to CBPAR projects and are composed of multiple key community partners. • Partners/individuals included the following: community leaders, local government representatives, educators and school administrators, nonprofit organization representatives, business owners and entrepreneurs, and faith-based organization leaders. The goals of the advisory board meetings were often stated as bringing diverse perspectives and expertise to the CBPAR research process. Specific discussions would include advising on research design, implementation, and dissemination of findings processes.	**11**
Photovoice	• An engagement method that empowers participants to use photography to capture and share their views and experiences with research teams. • Participants are typically provided with cameras to document their daily lives, challenges, and strengths within their communities, which are in-turn used to stimulate discussion and advocate for changes to address their community needs; helps to ensure that research outcomes are grounded in the lived realities of communities.	**7**

Broad categories of the primary engagement methods used to engage marginalized pediatric populations in the studies. Multiple studies utilized multiple primary engagement methodologies

**Table 5:** Levels of engagement

Area of Engagement	Number of Studies	Studies
Study design and development	34	Blanchet 2017, Cruz 2014, Garcia 2012, Flicker 2008, Garwick 2010, Green 2016, Jadwin-Cakmak 2019, Javier 2010, Katz-Wise 2019, Langdon 2016, Lesser 2013, Liu 2011, Livingood 2017, Martin 2010, McCalman 2020, Merves 2015, Muhwezi 2019, Renzaho 2015, Rocha 2022, Shea 2013, Shelef 2016, Shikako-Thomas 2016, Singer 2022, Tanjasiri 2011, Teela 2022, Thomas 2009, Ungar 2015, Valdez 2020, Williamson 2022, Willis 2020, Willis 2016, Yan 2021, Callejas 2022
Recruitment	24	Blanchet 2017, Cruz 2014, Flicker 2008, Garwick 2010, Jadwin-Cakmak 2019, Javier 2010, Katz-Wise 2019, Langdon 2016, Liu 2011, Livingood 2017, Merves 2015, Muhwezi 2019, Renzaho 2015, Rocha 2022, Savage 2006, Shea 2013, Shikako-Thomas 2016, Singer 2022, Tanjasiri 2011, Tiwari 2014, Vindrola-Padros 2016, Willis 2016, Willows 2016, Yan 2021
Data collection	9	Green 2016, Livingood 2017, Rocha 2022, Singer 2022, Traube 2013, Ungar 2015, Vindrola-Padros 2016, Willis 2016, Yan 2021
Data interpretation	12	Katz-Wise 2019, Liu 2011, Livingood 2017, Rocha, Savage 2006, Shelef 2016, Singer 2022, Ungar 2015, Valdez 2020, Willis 2016, Yan 2021, Yonas 2013
Data dissemination	17	Garwick 2010, Katz-Wise 2019, Langdon 2016, Liu 2011, Livingood 2017, Renzaho 2015, Rocha 2022, Shea 2013, Singer 2022, Tanjasiri 2011, Thomas 2009, Valdez 2020, Vindrola-Padros 2016, Willis 2016, Willows 2016, Yan 2021, Yonas 2013

Details on the degree of involvement of the engaged marginalized pediatric populations in the studies, broken done by phases of research

**Table 6:** Study evaluations of CBPR methodologies utilized

Method of Assessment	Description and Examples	Number of Studies	Studies
None	These studies did not describe undertaking evaluation of their CBPAR methodology and/or process.	21	Blanchet 2017, Cruz 2014, Cui 2019, Garcia 2012, Garwick 2010, Merves 2015, Raanaas 2020, Renzaho 2015, Rocha 2023, Savage 2006, Shalowitz 2009, Shelef 2016, Simpson 2022, Teela 2022, Thomas 2009, Vaughn 2013, Vindrola-Padros 2016, Callejas 2022, Willows 2016, Yan 2021, Yonas 2013
Participant Assessment	**Surveys**: Participants were invited to complete brief, anonymous surveys about their experience in the CBPAR process (either at the end or throughout the process).	5	Flicker 2008, Javier 2010, Katz-Wise 2019, Langdon 2016, Shea 2013
	**Focus Groups/ Interviews**: Individual and focus group interviews with participants to collect feedback on the study process. These were also done in combination with knowledge translation/dissemination sessions (e.g. community report-back sessions) at the conclusion of the research study.	13	Flicker 2008, Green 2016, Katz-Wise 2019, Langdon 2016, Lesser 2013, Liu 2011, Livingood 2017, Muhwezi 2019, Singer 2022, Tanjasiri 2011, Traube 2013, Ungar 2015, Williamson 2022
Researcher Assessment	**Recruitment Statistics**: Purposeful collection of study statistics including participant recruitment and retention rates of individuals to participate in study development and partnership aspects.	7	Flicker 2008, Jadwin-Cakmak 2019, Katz-Wise 2019, Langdon 2016, Tanjasiri 2011, Tiwari 2014, Valdez 2021
	**Deliverables/Outcome Measurements**: These studies attempted to assess the “success of achieving the desired outcome” with respect to the research study objectives. They considered whether using CBPAR affected the overall research process and subsequently supported them in conducting a more effective study with regards to the primary study outcome objectives. Studies considered data such as amount and quality of photovoice data collected, number of participants who were recruited and retained from target communities to participate within the study.	7	Flicker 2008, Martin 2010, McCalman 2020, Muhwezi 2019, Valdez 2020, Willis 2016, Willis 2020

Approaches to evaluation of the CBPR methodologies employed in the studies included in the review. We outline how the research teams themselves tried to understand the effectiveness of their engagement methodologies

## Discussion

This scoping review aimed to explore the use of community-based participatory research (CBPR) methodologies in engaging vulnerable and marginalized pediatric populations in health research. Despite the potential of CBPR to foster equitable research practices and address health disparities, our findings reveal substantial variability in how CBPR principles are defined and operationalized, as well as critical gaps in partner engagement, evaluation, and the development of standardized frameworks and approaches in CBPR with these communities. As we reflect on the future of pediatric health equity research, these findings have significant implications for improving adoption and implementation of CBPR methodologies. Ultimately a further understanding of CBPR opportunities that engage marginalized pediatric populations can help researchers more effectively engage this group to advance their health outcomes. The discussion is also informed by the insights of our patient partner.

One of the significant findings of our review is the wide variability in CBPR approaches described in the various studies. CBPR’s foundational principles emphasize equitable partnerships, co-creation of knowledge, and shared ownership throughout the research process [16]. However, our review identified a range of interpretations and applications of these principles. Some variation is expected given the different target communities and cultural/geographic contexts of the studies, but as pediatric researchers it is important that we recognize the degree of variation and lack of shared terminology anddefinitions currently employed. To ensure rigorous, high quality studies that are able to advance health outcomes, it may be helpful to continue developing shared standards and common practices. We can also draw on best practices in implementation science to move this research field forward. While most studies involved partners in the design phase, fewer engaged them during other critical stages, including recruitment, data collection, analysis, and dissemination. Alarmingly, only 11% of studies included community partners across all phases of research. The extent to which CBPR studies that are focused on other populations involve community partners across all phases is not clear, but other reviews focused on patient engagement in research have noted limitations of the extent of patient involvement [17, 18]. This lack of comprehensive engagement raises concerns about the authenticity of CBPR efforts and highlights a recurring challenge: distinguishing between genuine participatory research and superficial applications of its principles. This is particularly critical, and often more challenging, when working with vulnerable and marginalized populations. Previous studies have documented concerns about tokenism in CBPR, where community involvement is symbolic rather than substantive, potentially leading to exploitation and harm to vulnerable populations [19, 20]. Ensuring adherence to authentic CBPR principles requires the establishment of clearer definitions and standards to guide both researchers and community partners.

Another important and critical finding of this review was the limited evaluation of CBPR methodologies. This is particularly important given these studies engaged marginalized pediatric populations and that CBPR continues to be an emerging methodological field amongst this group. Nearly half (46%) of the studies did not formally assess their engagement processes. Among those that did, evaluation methods varied widely, with no consistent metrics or frameworks to measure effectiveness. Most evaluations focused on intervention outcomes rather than the success of engagement strategies, limiting the ability to refine methodologies or assess their impact on marginalized populations. It is critical to recognize that even if a general CBPR methodology and approach works in one population, it may not translate well to others without significant adaptation [16]. The absence of evaluation data impedes efforts to establish evidence-based best practices for CBPR. Without robust evaluation, it is difficult to determine whether participatory approaches genuinely empower communities, improve research outcomes, or address health inequities [21]. This aligns with findings from Wallerstein and colleagues [22], who emphasized that developing systematic evaluation frameworks is essential for enhancing the rigour and impact of CBPR.

Our review objectives also focused on identifying challenges in engaging marginalized pediatric populations. Engaging these patients and families in CBPR presents unique challenges rooted in historical, structural, and logistical barriers [23]. These obstacles, including systemic inequities, historical exploitation, and lack of representation in research, influence recruitment, retention, and the overall success of CBPR initiatives [23]. We will explore a number of these in detail below:

Historical Mistrust and Structural Inequities: A significant barrier is the deep mistrust of research institutions among marginalized communities, stemming from unethical practices like the Tuskegee Syphilis Study [24] and the exploitation of Indigenous knowledge [25]. This mistrust, compounded by caregivers’ protective instincts, often results in hesitancy to participate. Families may fear that research could perpetuate inequities or stigmatization. Addressing this requires culturally sensitive approaches, sustained trust-building efforts, and transparent communication emphasizing reciprocal benefits [26, 27].

Logistical Barriers: Marginalized families often face logistical obstacles, such as transportation issues, language barriers, and limited access to technology. These challenges disproportionately affect participation. Flexible research designs, such as virtual participation options, multilingual staff, and support for transportation or childcare, can mitigate these barriers. Researchers should involve community members in identifying solutions to ensure relevance and accessibility. An important consideration includes working with institutional ethics boards to create procedures that support the flexibility required to conduct true community engaged research. This may involve supporting more community engagement with institutional ethics boards to support the mission of protecting patients and the public while furthering partnerships that can truly address important research objectives.

Recruitment and Representation: Traditional recruitment methods often fail to resonate with vulnerable and marginalized populations. Effective strategies include leveraging relationships with trusted community organizations, such as youth groups, schools, and faith-based organizations, which help establish credibility and reach underrepresented groups. Recruitment approaches must also account for intersectional identities (e.g., low-income LGBTQ + youth) to ensure diverse participation [27].

Ethical Considerations and Power Dynamics: Ethical challenges and power imbalances are significant concerns in CBPR [28]. Marginalized communities are often excluded from decision-making, reducing their role to passive subjects. Addressing this requires equitable participation, shared decision-making, and transparent communication at every stage of research. Co-leadership models and ensuring data ownership by communities are critical for ethical, respectful CBPR practices [29]. For pediatric populations, age-appropriate consent and assent processes are essential [30].

Building Long-Term Partnerships: Short-term research projects often fail to establish trust-based relationships with marginalized communities, leading to skepticism about future efforts [31]. Long-term partnerships that include sustained engagement, capacity-building initiatives, and community advisory boards can address this challenge, fostering deeper collaboration and trust.

Cultural Sensitivity and Adaptation: Cultural sensitivity is essential but often challenging to implement. Researchers must respect diverse norms and values while avoiding assumptions about communities. For example, engaging Indigenous youth may require incorporating traditional knowledge and ceremonies [32], while newcomer families may need approaches that address cultural stigmas relevant to their specific culture [33]. Partnering with community leaders is crucial to ensuring that methodologies are both respectful and culturally relevant [23]. 

Implications for CBPR with Marginalized Pediatric Populations.

The findings of this review underscore several key learnings that have implications for future CBPR efforts with marginalized pediatric populations:

1. Trust-Building as a Foundation: Addressing historical mistrust requires deliberate and sustained efforts to build relationships with marginalized communities. Partnering with trusted local organizations and engaging community members early in the research process are essential strategies for fostering trust and collaboration.
2. Capacity Building for Sustainable Engagement: Providing community members with the resources and skills needed to actively participate in research empowers them to take ownership of the process. Capacity-building initiatives not only enhance the quality of engagement but also ensure that research outcomes are grounded in the lived realities of the participants.
3. Flexible Frameworks with Quality Standards that support strong community engagement and adaptability: The development of adaptable guidelines and evaluation frameworks can help bring elements of standardization to CBPR methodologies so that we ensure the research is truly community driven and rigorous. At the same time we must recognize the importance of flexibility allowing for cultural sensitivity and contextual relevance. Such frameworks should be co-created with community partners to ensure they address the unique needs of diverse populations. They also can serve as initial ‘starting points’ for research teams who wish to begin their journey in developing CBPR studies with marginalized pediatric populations.
4. Prioritizing Methodological Evaluation: Establishing robust evaluation practices is crucial for assessing the effectiveness of CBPR methodologies. Future research should focus on developing and implementing standardized metrics to evaluate both engagement processes and health outcomes, ensuring the broader applicability of CBPR methods.
5. Intersectional Approaches: Recognizing the intersecting identities and experiences of marginalized pediatric populations is critical for designing inclusive CBPR efforts. Intersectional frameworks can help researchers address overlapping inequities and ensure that research methods are tailored to the specific needs of diverse communities.

### Limitations

While this review provides valuable insights, it is important to acknowledge its limitations. First, restricting the inclusion criteria to English-language publications may have excluded relevant studies from non-English-speaking regions. Second, the variability in how CBPR principles were defined and described across studies presented challenges in coding and analyzing data consistently. Some studies lacked clarity in their methodological reporting, limiting our ability to assess the depth and quality of community engagement. Despite these limitations, this review highlights critical gaps and opportunities in the field, laying a foundation for future research.

## Conclusion

This scoping review highlights both the promise and the challenges of using CBPR methodologies to engage marginalized pediatric populations in health research. While CBPR has the potential to address health disparities and advance equity, its application in pediatric settings remains inconsistent and under-evaluated. By addressing the gaps identified in this review—such as the need for comprehensive engagement, robust evaluation, and standardized frameworks—researchers can enhance the impact and equity of CBPR, ultimately contributing to more inclusive and effective health interventions.

Future efforts must center the voices and experiences of marginalized populations, ensuring that CBPR is not only a method but a pathway to meaningful change. Through sustained collaboration, cultural humility, and a steadfast commitment to equity, the field of pediatric health research can advance toward a more inclusive and just future.

## Appendix

Search strategy example:

***Medline***

Set 1 Research.

((*Biomedical Research/ or *Health planning/) and (*Patient Participation/)) or exp Community-Based Participatory Research/.

or.

((community-based or patient engagement* or patient participation*) adj2 (approach* or method* or model$ or framework$ or barrier* or facilitat*)).tw, kf.

((PPI or “patient and public” or user$ or patient$ or family or families or parent* or grandparent* or guardian$ or carer$ or caregivers$ or “Community-Based” or “patient-orient*” or CBPR or community or meaningful*) adj (engage* or participat* or involv* or inclusion or includ* or partner* or health plann* or health services plan* or healthcare plann*)).tw, kf.

Set 2 barriers to access.

*"Communication Barriers”/ or *language/ or *informed consent/ or (“Limited English” or “language proficien*” or “English proficien*” or “Language barrier*” or “Communication Barrier*” or “Informed Consent” or translator$).tw, kf.

or.

(translat* adj2 service$).tw, kf.

Or.

(barrier* adj2 (sampling or recruitment or participation or retention or access or scope)).tw, kf.

*Health Services Accessibility/ or Health Status Disparities/ or Healthcare Disparities/ or *Cultural Deprivation/ or *Poverty/ or racism/ or *equity/.

or.

((system* or institutional* or structur* or attitude* or bias* or discriminat*) adj (Racis* or racial*)).tw, kf.

Or.

((social or socioeconomic or racial* or cultur* or ethnic* or wealth or income) adj (inequal* or unequal* or disparit* or inequit*)).tw, kf.

or.

Cultural Competency/ or (cultur* adj (safe* or competen* or sensitiv*)).tw, kf.

Set 3 Pediatrics.

Exp Child/ or exp Adolescent/ or exp infant/ or (child* or “child health” or adolescen* or infan* or baby or babies or neonat* or newborn$ or new born$ or toddler$ or teen* or teen-age* or preschool* or pre school* or school age* or youth$ or juvenile* or highschool* or boy$ or girl$ or p$ediatric*).tw, kf.

Set 4 marginalized populations.

*Vulnerable Populations/ or Indigenous Canadians/ or Health Services, Indigenous/ or *Social Marginalization/.

(African American* or black$ or Amish or Arab* or Asian American* or pacific islander$ or Indigenous People$ or Jew$ or jewish or Roma or “Sexual and Gender Minorit*” or Intersex Person$ or Transgender* or “native american*” or “native Canadian*” or “First Nations” or Metis or inuit or Innu or “Alaskan native*” or “North American Indian$” or rural).tw, kf.

((Vulnerable or “hard-to-reach” or disadvantage* or marginali$ed or poor or “low income” or “low socioeconomic status” or “low SES” or immigrant$ or newcomer$ or new-comer$ or refugee* or minority or ethnic* or racial* or cultural minorit* or religious minorit* or equity-deserving or underrepresented or traditionally marginali$ed* or urban or disabilit* or disabl*) adj (population$ or group$ or person$ or people$ or individual$ or communit*)).tw, kf.

Or.

(represent* adj (health research or medical research)).tw, kf.

(Barrier$ adj4 (disadvantaged group$ or marginali$ed group$)).tw, kw, kf.

**Table:** 

#	Searches	Results
1	((*Biomedical Research/ or *Health planning/) and *Patient Participation/) or exp Community-Based Participatory Research/	6125
2	((community-based or patient engagement* or patient participation*) adj2 (approach* or method* or model$ or framework$ or barrier* or facilitat*)).tw, kf.	6431
3	((PPI or “patient and public” or user$ or patient$ or family or families or parent* or grandparent* or guardian$ or carer$ or caregivers$ or “Community-Based” or “patient-orient*” or CBPR or community or meaningful*) adj (engage* or participat* or involv* or inclusion or includ* or partner* or health plann* or health services plan* or healthcare plann*)).tw, kf.	134,438
4	or/1–3	144,371
5	*"Communication Barriers”/ or *language/ or *informed consent/ or (“Limited English” or “language proficien*” or “English proficien*” or “Language barrier*” or “Communication Barrier*” or “Informed Consent” or translator$).tw, kf.	88,750
6	(translat* adj2 service$).tw, kf.	297
7	(barrier* adj2 (sampling or recruitment or participation or retention or access or scope)).tw, kf.	6729
8	*Health Services Accessibility/ or Health Status Disparities/ or Healthcare Disparities/ or *Cultural Deprivation/ or *Poverty/ or racism/ or *equity/	97,184
9	((system* or institutional* or structur* or attitude* or bias* or discriminat*) adj (Racis* or racial*)).tw, kf.	2044
10	((social or socioeconomic or racial* or cultur* or ethnic* or wealth or income) adj (inequal* or unequal* or disparit* or inequit*)).tw, kf.	27,015
11	Cultural Competency/ or (cultur* adj (safe* or competen* or sensitiv*)).tw, kf.	17,155
12	or/5–11	222,988
13	4 and 12	5718
14	exp Child/ or exp Adolescent/ or exp infant/ or (child* or “child health” or adolescen* or infan* or baby or babies or neonat* or newborn$ or new born$ or toddler$ or teen* or teen-age* or preschool* or pre school* or school age* or youth$ or juvenile* or highschool* or boy$ or girl$ or p$ediatric*).tw, kf.	4,689,714
15	13 and 14	1777
16	*Vulnerable Populations/ or Indigenous Canadians/ or Health Services, Indigenous/ or *Social Marginalization/	9843
17	(African American* or black$ or Amish or Arab* or Asian American* or pacific islander$ or Indigenous People$ or Jew$ or jewish or Roma or “Sexual and Gender Minorit*” or Intersex Person$ or Transgender* or “native american*” or “native Canadian*” or “First Nations” or Metis or inuit or Innu or “Alaskan native*” or “North American Indian$” or rural).tw, kf.	590,167
18	((Vulnerable or “hard-to-reach” or disadvantage* or marginali$ed or poor or “low income” or “low socioeconomic status” or “low SES” or immigrant$ or newcomer$ or new-comer$ or refugee* or minority or ethnic* or racial* or cultural minorit* or religious minorit* or equity-deserving or underrepresented or traditionally marginali$ed* or urban or disabilit* or disabl*) adj (population$ or group$ or person$ or people$ or individual$ or communit*)).tw, kf.	127,181
19	(represent* adj (health research or medical research)).tw, kf.	4
20	(Barrier$ adj4 (disadvantaged group$ or marginali$ed group$)).tw, kw, kf.	12
21	or/16–20	687,627
22	13 and 21	1970
23	15 or 22	3124
24	(“23232779” or “24669751” or “25423241” or “29544486”).ui.	4
25	23 and 24	4
26	limit 23 to humans	2811
27	remove duplicates from 26	2800

***Updated search***

Ovid MEDLINE(R) and Epub Ahead of Print, In-Process, In-Data-Review & Other Non-Indexed Citations and Daily < 1946 to November 22, 2024>.

**Table:** 

#	Searches	Results
1	((*Biomedical Research/ or *Health planning/) and *Patient Participation/) or exp Community-Based Participatory Research/	7050
2	((community-based or patient engagement* or patient participation*) adj2 (approach* or method* or model$ or framework$ or barrier* or facilitat*)).tw, kf.	8271
3	((PPI or “patient and public” or user$ or patient$ or family or families or parent* or grandparent* or guardian$ or carer$ or caregivers$ or “Community-Based” or “patient-orient*” or CBPR or community or meaningful*) adj (engage* or participat* or involv* or inclusion or includ* or partner* or health plann* or health services plan* or healthcare plann*)).tw, kf.	163,829
4	or/1–3	175,941
5	*"Communication Barriers”/ or *language/ or *informed consent/ or (“Limited English” or “language proficien*” or “English proficien*” or “Language barrier*” or “Communication Barrier*” or “Informed Consent” or translator$).tw, kf.	100,527
6	(translat* adj2 service$).tw, kf.	363
7	(barrier* adj2 (sampling or recruitment or participation or retention or access or scope)).tw, kf.	9058
8	*Health Services Accessibility/ or Health Status Disparities/ or Healthcare Disparities/ or *Cultural Deprivation/ or *Poverty/ or racism/ or *equity/	107,112
9	((system* or institutional* or structur* or attitude* or bias* or discriminat*) adj (Racis* or racial*)).tw, kf.	3578
10	((social or socioeconomic or racial* or cultur* or ethnic* or wealth or income) adj (inequal* or unequal* or disparit* or inequit*)).tw, kf.	35,855
11	Cultural Competency/ or (cultur* adj (safe* or competen* or sensitiv*)).tw, kf.	20,685
12	or/5–11	257,015
13	4 and 12	7068
14	exp Child/ or exp Adolescent/ or exp infant/ or (child* or “child health” or adolescen* or infan* or baby or babies or neonat* or newborn$ or new born$ or toddler$ or teen* or teen-age* or preschool* or pre school* or school age* or youth$ or juvenile* or highschool* or boy$ or girl$ or p$ediatric*).tw, kf.	5,054,887
15	13 and 14	2119
16	*Vulnerable Populations/ or Indigenous Canadians/ or Health Services, Indigenous/ or *Social Marginalization/	10,772
17	(African American* or black$ or Amish or Arab* or Asian American* or pacific islander$ or Indigenous People$ or Jew$ or jewish or Roma or “Sexual and Gender Minorit*” or Intersex Person$ or Transgender* or “native american*” or “native Canadian*” or “First Nations” or Metis or inuit or Innu or “Alaskan native*” or “North American Indian$” or rural).tw, kf.	695,761
18	((Vulnerable or “hard-to-reach” or disadvantage* or marginali$ed or poor or “low income” or “low socioeconomic status” or “low SES” or immigrant$ or newcomer$ or new-comer$ or refugee* or minority or ethnic* or racial* or cultural minorit* or religious minorit* or equity-deserving or underrepresented or traditionally marginali$ed* or urban or disabilit* or disabl*) adj (population$ or group$ or person$ or people$ or individual$ or communit*)).tw, kf.	152,019
19	(represent* adj (health research or medical research)).tw, kf.	5
20	(Barrier$ adj4 (disadvantaged group$ or marginali$ed group$)).tw, kw, kf.	15
21	or/16–20	811,269
22	13 and 21	2510
23	15 or 22	3873
24	limit 23 to humans	3421
25	limit 24 to dt = 20,220,823–20,241,125	561
26	limit 24 to ed = 20,220,823–20,241,125	622
27	limit 24 to ez = 20,220,823–20,241,125	561
28	or/25–27	622
29	remove duplicates from 28	609

## Electronic supplementary material

Below is the link to the electronic supplementary material.

Supplementary Information 1

## Data Availability

Data is provided within the manuscript.Additional data is avaiable on request.
