# Supplementary material for: Methods for engaging vulnerable and marginalized children through community based participatory research: a scoping review
Source: Res Involv Engagem. 2025 Aug 27;11:104. doi: 10.1186/s40900-025-00783-3 (PMC12392517; doi:10.1186/s40900-025-00783-3)
Supplement: Supplementary file 1 — Supplementary Information 1 [file 40900_2025_783_MOESM1_ESM.docx]

GRIPP2 Short Form Checklist

| **Section and topic** | **Item** | **Reported on page No** |
| --- | --- | --- |
| 1: Aim | Report the aim of PPI in the study | Page 10 |
| 2: Methods | Provide a clear description of the methods used for PPI in the study | Page 10 |
| 3: Study results | Outcomes—Report the results of PPI in the study, including both positive and negative outcomes | N/A |
| 4: Discussion and conclusions | Outcomes—Comment on the extent to which PPI influenced the study overall. Describe positive and negative effects | Page 25 |
| 5: Reflections/critical perspective | Comment critically on the study, reflecting on the things that went well and those that did not, so others can learn from this experience | N/A |

This submission is a review article.
